# Connecting a disconnected trial network with a new trial: optimizing the estimation of a comparative effect in a network meta-analysis

**DOI:** 10.1186/s12874-023-01896-7

**Published:** 2023-04-03

**Authors:** Lauren McKeen, Paul Morris, Chong Wang, Max D. Morris, Annette M. O’Connor

**Affiliations:** 1 Department of Statistics, Iowa State University, Ames, USA; 2 Department of Veterinary Diagnostic and Production Animal Medicine, Iowa State University, Ames, USA; 3 Department of Large Animal Clinical Sciences, Michigan State University, East Lansing, USA

**Keywords:** Network meta-analysis, Clinical trial design, Evidence synthesis

## Abstract

**Background:**

In network meta-analysis, estimation of a comparative effect can be performed for treatments that are connected either directly or indirectly. However, disconnected trial networks may arise, which poses a challenge to comparing all available treatments of interest. Several modeling approaches attempt to compare treatments from disconnected networks but not without strong assumptions and limitations. Conducting a new trial to connect a disconnected network can enable calculation of all treatment comparisons and help researchers maximize the value of the existing networks. Here, we develop an approach to finding the best connecting trial given a specific comparison of interest.

**Methods:**

We present formulas to quantify the variation in the estimation of a particular comparative effect of interest for any possible connecting two-arm trial. We propose a procedure to identify the optimal connecting trial that minimizes this variation in effect estimation.

**Results:**

We show that connecting two treatments indirectly might be preferred to direct connection through a new trial, by leveraging information from the existing disconnected networks. Using a real network of studies on the use of vaccines in the treatment of bovine respiratory disease (BRD), we illustrate a procedure to identify the best connecting trial and confirm our findings via simulation.

**Conclusion:**

Researchers wishing to conduct a connecting two-arm study can use the procedure provided here to identify the best connecting trial. The choice of trial that minimizes the variance of a comparison of interest is network dependent and it is possible that connecting treatments indirectly may be preferred to direct connection.

## Background

Network meta-analysis (NMA) enables estimation of comparative effects of treatments that are directly connected as well as those that are indirectly connected. Through such direct or indirect comparisons, researchers and clinicians are able to obtain comparisons of treatments available in an entire evidence network, where a network is defined as a collection of trials that compare some number of treatments for a given clinical outcome [1]. Networks are often referred to as graphs, which consist of nodes, e.g., treatments, and edges (or links) that represent direct comparisons between treatments. The studies included in a NMA of treatments are ideally randomized controlled trials identified as a result of a systematic review such that the included trials are consistent with the assumptions of an NMA. The transitivity assumption states that each subject in a trial must have been eligible for enrollment in all other trials. If this assumption is violated, then the estimates of the direct and indirect comparative effects may not be valid. Further, the consistency assumption states that pairwise comparisons between treatments must be able to be written as a function of the baseline treatment. Again, this assumption is vital for proper estimation of the direct and indirect comparative effects.

The evidence base for treatments is often driven by the interests of individual researchers and funding agencies that fail to consider how to maximize the value of the entire evidence base. As a result, networks of trials can be disconnected, in which there is neither a direct or indirect route to compare all treatments. Conversely, a connected network is one in which there is a path, or edge, “linking”, or connecting, every treatment to all others. Disconnected networks may arise when there is no common standard of treatment or when there are many available treatments [2]. Such networks pose difficulties when researchers wish to make comparisons between all possible treatments. Similarly, the issue of disconnected experimental design with respect to treatments has been studied [3], where the focus has been on checking and avoiding disconnected treatments in one single experiment.

There are several proposed approaches for dealing with disconnected networks in NMA. One approach relies on the use of non-randomized evidence to connect the networks [4]. The use of non-randomized evidence, often referred to as real world evidence (RWE), assumes that the expected response to a control treatment is constant between historical studies and the randomized studies [5]. This assumption is thought to be both unlikely and associated with bias [1]. Component network meta analysis (CNMA) has been proposed as an alternative [1]. While CNMA addresses the issues of using RWE, in terms of not relying on the aforementioned assumptions, the networks must be of a certain form. That is, the treatments must consist of common treatment components occurring in both networks (i.e., disconnected networks can be bridged together only if the networks are made up of multi-component treatments that are common to each network). There are both additive and two-way interaction models for CNMA, but in order to connect a disconnected network, the authors note that at least some treatments must consist of components and the sub-networks need a “sufficient” number of common components. Another modeling approach to analyze disconnected networks is through random baseline effects [6]. This method has been found to be appropriate for two example data sets, but the authors note that there is a risk for the assumptions regarding the normality and exchangeability of the baseline treatments effects to be violated in other data sets. There are also several population adjustment methods for disconnected networks proposed [7] and in the case of dose-response modeling, there are methods to make comparisons between treatments belonging to disconnected sub-networks [8]. All of the methods mentioned here show motivation for connecting disconnected networks, but all are limited in their own ways.

Given the strong reliance on assumptions in the aforementioned approaches, researchers may decide to design new studies that connect a disconnected network. While there are many methods that discuss how to design a trial within an existing connected network [9–13], we are unable to identify literature for designing a connecting trial for a disconnected network. In this paper, we formalise an approach to connecting two components of a disconnected network, which we refer to as sub-networks, with a new two-arm trial, based on an approach that minimizes the variance of a comparative effect estimation between two treatments of interest. The sub-networks themselves must meet the aforementioned assumptions of NMA. After deriving variance formulas for a particular effect size estimate under a connecting trial, we propose a straightforward computational procedure to guide researchers conducting a trial. We confirm these results through a simulation study. We conclude that given a comparison of treatments, the best connecting two-arm trial in terms of minimizing the estimation variation is network dependent and can be found through a straightforward computational procedure.

## Methods

To consider how to best connect two sub-networks from a disconnected network, we focus on minimizing the variance of a specific comparative effect size between two treatments, which we call a comparison of interest (COI). Here, the term treatment is generic and could refer to any active intervention or a placebo. Our goal is to form a connected network, where a connected network is formally defined as one in which, for any two treatments (A,B), there exists an ordered sequence of treatments ($t_1,t_2,\ldots ,t_k$), k$\le$T-2, where T is the total number of treatments, such that:

- Treatments A and $t_1$ are both included in one or more trials,
- Treatments $t_1$ and $t_2$ are both included in one or more trials,
- ......,
- Treatments $t_k$ and B are both included in one or more trials.

For treatments (A,B) that are included in the same trial, the comparison is direct and the ordered sequence is empty. Within such a network, the difference in effects between any two treatments can be evaluated through the path “linking”, or connecting, the treatments. In this section, we establish formulas for the variation of a particular COI under several scenarios. The organization of the section is as follows. We review the variance estimation of a comparative effect size in a traditional two-arm trial. We then review general notation and variance estimation of a comparative effect under a fixed effects NMA model. Next, we establish the notation and properties of disconnected networks. Last, we derive variance formulas under the connection of two disconnected sub-networks using a two-arm trial.

### Estimating the variance of a comparison of interest in a traditional two-arm trial

Suppose we conduct a two-arm trial with a binary outcome using treatments A and B, with a total fixed sample size *n* such that $n_A + n_B = n$. Let $r_i$ denote the number of events in the subjects belonging to treatment group *i*, $i \in \{A,B\}$. Then the number of events $r_i$ follows a binomial distribution; that is, $r_i \sim \text {binomial}(n_i,p _i)$, where $p _i$ is the probability of an event occurring in treatment *i*. Through a Bernoulli generalized linear model, we have

1 $$\begin{aligned} \text {log}(\frac{p _i}{1-p _i}) = \beta _0 + \beta _1 I_{(i=\text {B})}. \end{aligned}$$

Here, $\beta _0$ is the log-odds of the probability of an event occurring in subjects in treatment group A, and $\beta _1$ is the log-odds ratio of treatment B to treatment A. We can estimate the coefficients $\beta _0$ and $\beta _1$ using a maximum likelihood approach and obtain the information matrix. The comparative effect of treatment B to A is of interest ($\hat{\beta _1}$), and it follows that

2 $$\begin{aligned} \text {Var}(\hat{\beta _1}) = \frac{1}{n_Ap_A(1-p_A)} + \frac{1}{n_Bp_B(1-p_B)}. \end{aligned}$$

Let $\text {Var}(\hat{\beta _1}) := \sigma ^2_{A,B}$ represent the estimated within-trial variance for the comparative effect size of treatment B to A. Note that in the context of NMA, $\beta _1$ is written as $\mu _{AB}$. We will utilize this derivation in our proposed procedure for connecting two disconnected sub-networks.

### Fixed effects NMA model: notation and estimating the variance of a COI

Now, consider a network of *T* treatments with *J* studies, and $n_j$ arms in the $j^{th}$ study. Let $\varvec{\mu }_b = (\mu _{AB}, \mu _{AC},\mu _{AD}, \ldots )'$ be the vector of comparative effect parameters of all treatments to the baseline treatment A. This is called the vector of basic parameters.

Let $\varvec{y}_j$ denote the observed comparative effect size for the $j^{th}$ study, $\varvec{y}_j = (y_{j, 1}, \ldots , y_{j, n_{j -1}})^{\prime }$, and $\varvec{y} = (\varvec{y}_1^{\prime }, \ldots , \varvec{y}_J^{\prime })^{\prime }.$ Let $\varvec{\mu }_j$ be the vector of comparative effect sizes for the $j^{th}$ study, $\varvec{\mu }_j = (\mu _{j, 1}, \ldots , \mu _{j, n_{j -1}})^{\prime }$, and $\varvec{\mu } = (\varvec{\mu }_1^{\prime }, \ldots , \varvec{\mu }_J^{\prime })^{\prime }.$ Then we have

3 $$\begin{aligned} \varvec{y}_j = \varvec{\mu }_j + \varvec{\epsilon }_j, j = 1,\ldots ,J, \end{aligned}$$

where $\varvec{\epsilon }_j$ is assumed to be normally distributed and independent across studies with covariance $\varvec{S}_j$ corresponding to the estimated within-trial variances. The distribution of $\varvec{y}$ is then MVN($\varvec{\mu }$, $\varvec{S}$), where $\varvec{S}$ is a block diagonal matrix with each block $\varvec{S}_j$. Since $\varvec{\mu }$ is a linear combination of $\varvec{\mu }_b$, it can be written as $\varvec{\mu } = \varvec{X}\varvec{\mu }_b$, where $\varvec{X}$ is the design matrix of size $\sum _{j=1}^{J} n_j \times (T-1)$. The distribution of $\varvec{y}$ is then MVN($\varvec{X}\varvec{\mu }_b$, $\varvec{S})$. The maximum likelihood estimate of $\varvec{\mu _b}$ and its variance are

4 $$\begin{aligned} \hat{\varvec{\mu }}_b = (\varvec{X}^{\prime }\varvec{S}^{-1}\varvec{X})^{-1}\varvec{X}^{\prime }\varvec{S}^{-1}\varvec{y}, \nonumber \\ \text {Var}(\hat{\varvec{\mu }}_b) = (\varvec{X}^{\prime }\varvec{S}^{-1}\varvec{X})^{-1}. \end{aligned}$$

Similar notation and derivations are provided below for disconnected networks.

### Notation and properties of disconnected networks

Suppose that the network of studies presented above is actually composed of *K* disconnected sub-networks. We can rewrite the vector of basic parameters as $\varvec{\mu }_b = (\mu _{AB}, \mu _{AC}, \mu _{AD}, \ldots )' = (\varvec{\mu }_{D1}', \varvec{\mu }_{D2}', \ldots , \varvec{\mu }_{DK}')'$, where $\varvec{\mu }_{Dk}'$ is the sub-vector corresponding to the basic parameters for treatments in the (disconnected) sub-network *k* compared to the overall baseline treatment, A.

Since the *K* sub-networks are not connected, we can rewrite the design matrix $\varvec{X}$ and variance matrix $\varvec{S}$ as block diagonal matrices corresponding to the components from the *K* sub-networks, denoted by the subscripts *Dk*, for $k = 1,\ldots ,K$:

5 $$\begin{aligned} \varvec{y} = \left[ \begin{array}{c} \varvec{y}_{D1}\\ \varvec{y}_{D2}\\ \vdots \\ \varvec{y}_{DK}\\ \end{array}\right] = \left[ \begin{array}{cccc} \varvec{X}_{D1} &{} 0 &{} \dots &{} 0\\ 0 &{} \varvec{X}_{D2} &{} \dots &{} 0\\ \vdots &{} \vdots &{} \ddots &{} \vdots \\ 0 &{} 0 &{} \dots &{} \varvec{X}_{DK} \end{array}\right] \left[ \begin{array}{c} \varvec{\mu }_{D1}\\ \varvec{\mu }_{D2}\\ \vdots \\ \varvec{\mu }_{DK} \end{array}\right] + \varvec{\epsilon }, \end{aligned}$$

where

$$\begin{aligned} cov(\varvec{\epsilon }) = \varvec{S} = \left[ \begin{array}{cccc} \varvec{S}_{D1} &{} 0 &{} \dots &{} 0\\ 0 &{} \varvec{S}_{D2} &{} \dots &{} 0\\ \vdots &{} \vdots &{} \ddots &{} \vdots \\ 0 &{} 0 &{} \dots &{} \varvec{S}_{DK} \end{array}\right] \end{aligned}$$

and $\varvec{y}_{Dk}$ are the observed comparative effects sizes for all studies in the *k*th sub-network.

Then we have

6 $$\begin{aligned} \hat{\varvec{\mu }}_{b}&= \left[ \begin{array}{c} (\varvec{X}_{D1}^{\prime }\varvec{S}_{D1}^{-1}\varvec{X}_{D1})^{-}\varvec{X}_{D1}^{\prime }\varvec{S}_{D1}^{-1}\varvec{y}_{D1}\\ (\varvec{X}_{D2}^{\prime }\varvec{S}_{D2}^{-1}\varvec{X}_{D2})^{-}\varvec{X}_{D2}^{\prime }\varvec{S}_{D2}^{-1}\varvec{y}_{D2}\\ \vdots \\ (\varvec{X}_{DK}^{\prime }\varvec{S}_{DK}^{-1}\varvec{X}_{DK})^{-}\varvec{X}_{DK}^{\prime }\varvec{S}_{DK}^{-1}\varvec{y}_{DK} \end{array}\right] ,\nonumber \\ \text {Var}(\hat{\varvec{\mu }}_{b})&= \left[ \begin{array}{cccc} (\varvec{X}_{D1}^{\prime }\varvec{S}_{D1}^{-1}\varvec{X}_{D1})^{-} &{} 0 &{} \dots &{} 0\\ 0 &{} (\varvec{X}_{D2}^{\prime }\varvec{S}_{D2}^{-1}\varvec{X}_{D2})^{-} &{} \dots &{} 0\\ \vdots &{} \vdots &{} \ddots &{} \vdots \\ 0 &{} 0 &{} \dots &{} (\varvec{X}_{DK}^{\prime }\varvec{S}_{DK}^{-1}\varvec{X}_{K})^{-} \end{array}\right] . \end{aligned}$$

With this notation, there are $T-1$ basic parameters. The $\text {rank}(\varvec{X}) = T - K$, where *K* is the number of disconnected sub-networks. Thus, $\hat{\varvec{\mu }}_b$ is not unique. Further, we propose the following lemma with regards to disconnected sub-networks.

#### Lemma 1

A set of *K* sub-networks are disconnected if and only if the design matrix for the entire network can be written in the form

$$\begin{aligned} \left[ \begin{array}{ccccc} \varvec{X}_{D1} &{} 0 &{} 0 &{} \dots &{} 0\\ 0 &{} \varvec{X}_{D2} &{} 0 &{} \dots &{} 0\\ 0 &{} 0 &{} \varvec{X}_{D3} &{} \dots &{} 0\\ \vdots &{} \vdots &{} \vdots &{} \ddots &{} \vdots \\ 0 &{} 0 &{} 0 &{} \dots &{} \varvec{X}_{DK} \end{array}\right] , \end{aligned}$$

with $$\varvec{\mu }_b = \left( \varvec{\mu }_{D1}, \varvec{\mu }_{D2}, \varvec{\mu }_{D3}, \ldots \varvec{\mu }_{DK}\right) '$$.

#### Proof

$\Leftarrow$ The design matrix is written so that each block corresponds to a sub-network, *Dk* for $k = 1, \ldots , K$. That is, estimates of comparative effect sizes from any given sub-network *Dk* can be written as linear combinations of parameters unique to sub-network *Dk*. Any sub-network *Dk* then depends solely on its own parameters and not on parameters from any other network. Thus, it follows that the entire network must be disconnected.

$\Rightarrow$ This follows directly from the setup above.   

With the aforementioned general notation and properties of disconnected networks, we can now consider variance estimation of a comparative effect size of interest when connecting two disconnected sub-networks with a two-arm trial.

### Estimating the variance of a comparison of interest under a new connecting two-arm trial

Consider a special case of the disconnected sub-networks above. That is, suppose that there are only $K=2$ disconnected sub-networks. Researchers wish to connect these two disconnected sub-networks with the goal of estimating a specific comparative effect size, or COI, as precisely as possible.

We first consider the case when the connecting trial is also the COI. We define this as a direct trial. Intuitively, all of the information about the comparison should be captured by the new observed trial data, so the variance of the comparison is the new within-trial variance. That is, the connecting trial encompasses all of the evidence for the comparison as the variance of the comparison of interest is the variance of the estimate of the comparative effect from the connecting trial. We develop this idea more formally below.

Suppose that we have two existing, disconnected sub-networks, with network one consisting of treatments $t_{11}, t_{12}, \ldots , t_{1m_1}$ and network two consisting of treatments $t_{21}, t_{22},\ldots , t_{2m_2}$. Without loss of generality, consider $t_{11}$ as the overall baseline treatment. We write the network model as:

7 $$\begin{aligned} \left[ \begin{array}{c} {\textbf {y}}_{D1}\\ {\textbf {y}}_{D2}\\ \end{array}\right]&= \left[ \begin{array}{cc} \varvec{X}_{D1} &{} \varvec{0}\\ \varvec{0} &{} \varvec{X}_{D2} \end{array}\right] \left[ \begin{array}{c} \varvec{\mu }_{D1}\\ \varvec{\mu }_{D2}\\ \end{array}\right] + \varvec{\epsilon }, \end{aligned}$$

where

$$\begin{aligned} cov(\varvec{\epsilon })&= \left[ \begin{array}{cc} \varvec{S}_{D1} &{} \varvec{0} \\ \varvec{0} &{} \varvec{S}_{D2} \end{array}\right] , \end{aligned}$$

and $\varvec{\mu }_{D1}, \varvec{\mu }_{D2}$ correspond to the basic parameters with respect to the overall baseline treatment, $t_{11}$. Connecting the two sub-networks with a study including the baseline treatment $t_{11}$, say with treatment $t_{21}$, gives us the model formulation:

8 $$\begin{aligned} \left[ \begin{array}{c} {\textbf {y}}_{D1}\\ {\textbf {y}}_{D2}\\ y_{t_{11},t_{21}} \end{array}\right]&= \left[ \begin{array}{ccc} \varvec{X}_{D1} &{} \varvec{0} &{} \varvec{0}\\ \varvec{0} &{} \varvec{X}_{D2_1} &{} \varvec{X}_{D2_2}\\ \varvec{0} &{} 1 &{} \varvec{0} \end{array}\right] \left[ \begin{array}{c} \varvec{\mu }_{D1}\\ \mu _{t_{11},t_{21}}\\ \varvec{\mu }_{D2}^{*}\\ \end{array}\right] + \varvec{\epsilon }, \end{aligned}$$

where

$$\begin{aligned} cov(\varvec{\epsilon })&= \left[ \begin{array}{ccc} \varvec{S}_{D1} &{} \varvec{0} &{} \varvec{0}\\ \varvec{0} &{} \varvec{S}_{D2} &{} \varvec{0}\\ \varvec{0} &{} \varvec{0} &{} \sigma ^{2}_{t_{11}, t_{21}} \end{array}\right] . \end{aligned}$$

Here, $y_{t_{11},t_{21}}$ is the data from the new connecting trial and $\sigma ^{2}_{t_{11}, t_{21}}$ is the new within-trial variance from the connecting trial. We partition $\varvec{X}_{D2}$ column-wise into $\varvec{X}_{D2_1}$ and $\varvec{X}_{D2_2}$ and partition $\varvec{\mu }_b$ such that the comparative effect size of interest is isolated. Then,

9 $$\begin{aligned} \text {Var}\left( \hat{\varvec{\mu }}_{D1}, \hat{\mu }_{t_{11},t_{21}}, \hat{\varvec{\mu }}_{D2}^{*}\right)&= \left[ \begin{array}{cc} (\varvec{X}_{D1}^{\prime }\varvec{S}_{D1}^{-1}\varvec{X}_{D1})^{-1} &{} \varvec{0}\\ \varvec{0} &{} \left( (\varvec{X}_{D2}^{\prime }\varvec{S}_{D2}^{-1}\varvec{X}_{D2}) + \left[ \begin{array}{cc} \sigma ^{2^{-1}}_{t_{11}, t_{21}} &{} \varvec{0} \\ \varvec{0} &{} \varvec{0} \end{array}\right] \right) ^{-1} \end{array}\right] . \end{aligned}$$

From this we see that finding $\text {Var}(\hat{\mu }_{t_{11},t_{21}})$, which is the variance of our COI under this setup, relies on inverting the matrix

$$\begin{aligned} (\varvec{X}_{D2}^{\prime }\varvec{S}_{D2}^{-1}\varvec{X}_{D2}) + \left[ \begin{array}{cc} \sigma ^{2^{-1}}_{t_{11}, t_{21}} &{} \varvec{0} \\ \varvec{0} &{} \varvec{0} \end{array}\right] . \end{aligned}$$

We now show that the variance of the COI, i.e., $\text {Var}(\hat{\mu }_{t_{11},t_{21}})$, simplifies to $\sigma ^2_{t_{11},t_{21}}$.

#### Proof

In order to show that the $\text {Var}(\mu _{t_{11},t_{21}}) = \sigma ^2_{t_{11},t_{21}}$ when two disconnected sub-networks are connected using $t_{11}$ and $t_{21}$, we use the following facts:

1. Given a block matrix $$\varvec{M} = \left[ \begin{array}{cc} \varvec{E} &{} \varvec{F}\\ \varvec{G} &{} \varvec{H} \end{array}\right]$$, where $\varvec{E},\varvec{F},\varvec{G},\varvec{H}$ are $n \times n, n \times m, m \times n, m \times m$ matrices with $\varvec{H}$ invertible,
  $$\begin{aligned} \text {det}(\varvec{M}) = \text {det}(\varvec{E}-\varvec{F}\varvec{H}^{-1}\varvec{G})\text {det}(\varvec{H}). \end{aligned}$$
  and
  $$\begin{aligned} \varvec{M}^{-1} = \left[ \begin{array}{cc} (\varvec{E}-\varvec{F}\varvec{H}^{-1}\varvec{G})^{-1} &{} -(\varvec{E}-\varvec{F}\varvec{H}^{-1}\varvec{G})^{-1}\varvec{F}\varvec{H}^{-1}\\ -\varvec{H}^{-1}\varvec{G}(\varvec{E}-\varvec{B}\varvec{H}^{-1}\varvec{G})^{-1} &{} \varvec{H}^{-1}+\varvec{H}^{-1}\varvec{G}(\varvec{E}-\varvec{F}\varvec{H}^{-1}\varvec{G})^{-1}\varvec{F}\varvec{H}^{-1} \end{array}\right] . \end{aligned}$$
2. If the above $\varvec{E}$ is scalar,
  $$\begin{aligned} \text {det}(\varvec{M}) = (\varvec{E}-\varvec{F}\varvec{H}^{-1}\varvec{G})\text {det}(\varvec{H}). \end{aligned}$$
3. If the rank of a square matrix $\varvec{J}$ of size $n \times n$ is less than *n*, $\text {det}(\varvec{J}) = 0$.

Now, we can write

$$\begin{aligned}&(\varvec{X}_{D2}^{\prime }\varvec{S}_{D2}^{-1}\varvec{X}_{D2}) + \left[ \begin{array}{cc} \sigma ^{2^{-1}}_{t_{11}, t_{21}} &{} \varvec{0} \\ \varvec{0} &{} \varvec{0} \end{array}\right] \\&= \left[ \begin{array}{cc} \varvec{A} &{} \varvec{B}\\ \varvec{C} &{} \varvec{D} \end{array}\right] + \left[ \begin{array}{cc} \sigma ^{2^{-1}}_{t_{11}, t_{21}} &{} \varvec{0} \\ \varvec{0} &{} \varvec{0} \end{array}\right] \\&= \left[ \begin{array}{cc} \varvec{A} + \sigma ^{2^{-1}}_{t_{11}, t_{21}} &{} \varvec{B} \\ \varvec{C} &{} \varvec{D} \end{array}\right] , \end{aligned}$$

where $\varvec{D}$ is invertible and $\varvec{X}_{D2}^{\prime }\varvec{S}_{D2}^{-1}\varvec{X}_{D2}$ is not full rank, which follows directly from the aforementioned model parameterization for disconnected networks. By facts 1-3, we have that the (1, 1)th element of the inverse is

$$\begin{aligned}&= (\varvec{A} + \sigma ^{2^{-1}}_{t_{11}, t_{21}} - \varvec{B}\varvec{D}^{-1}\varvec{C})^{-1}\\&= (\varvec{A} - \varvec{B}\varvec{D}^{-1}\varvec{C} + \sigma ^{2^{-1}}_{t_{11}, t_{21}})^{-1}\\&= (0 + \sigma ^{2^{-1}}_{t_{11}, t_{21}})^{-1}\\&= \sigma ^{2}_{t_{11}, t_{21}}, \end{aligned}$$

which completes the proof.   

The results here are limited to the case when the COI is exactly the connecting trial (i.e., a direct trial). We now present variance formulas for indirect trials. We define a partially indirect connecting trial as one that involves exactly one of the two treatments in the COI, and a completely indirect trial involves neither of the two treatments in the COI.

Consider again two existing, disconnected sub-networks, with sub-network one consisting of treatments $t_{11}. t_{12}, \ldots , t_{1m_1}$ and sub-network two consisting of treatments $t_{21}, t_{22},\ldots , t_{2m_2}$, with $t_{11}$ as the overall baseline treatment. The two sub-networks are connected through a new trial including treatments $t_{11}$ and $t_{21}$ such that the within-trial variance is $\sigma ^2_{t_{11},t_{21}}$. We have shown above that $Var(\hat{\mu }_{t_{11},t_{21}}) = \sigma ^2_{t_{11},t_{21}}$. We will now show that for any COI $\mu _{t_{1i},t_{2j}}$ for $i = 2,\ldots ,m_1, j = 2,\ldots ,m_2$ the variance of the estimate of the COI can be written as the sum of $\sigma ^2_{t_{11},t_{21}}$ and additional variance terms from sub-networks. Note that the COI in this case is **not** obtained from the connecting trial.

#### Proof

To start, suppose we are interested in a comparison between the overall baseline $t_{11}$ and an arbitrary treatment from sub-network two, $t_{2j}, j = 2,\ldots ,m_2$. By independence and consistency assumptions [14], we have the following:

10 $$\begin{aligned} Var\left( \hat{\mu }_{t_{11},t_{2j}}\right)&= Var\left( \hat{\mu }_{t_{11},t_{21}} + \hat{\mu }_{t_{21},t_{2j}}\right) \nonumber \\&= \sigma ^2_{t_{11},t_{21}} + \sigma ^2_{t_{21},t_{2j,pooled}}, \end{aligned}$$

where $\sigma ^2_{t_{21},t_{2j,pooled}}$ is a pooled variance from the NMA analysis on sub-network 2. This is an example of a partially indirect connecting trial as defined earlier. If we are interested in a non-baseline comparison between treatments $t_{1i}$ and $t_{2j}$ ,$i = 2,\ldots ,m_1, j = 2,\ldots ,m_2$, it follows that

11 $$\begin{aligned} Var\left( \hat{\mu }_{t_{1i},t_{2j}}\right)&= Var\left( \hat{\mu }_{t_{11},t_{1i}} + \hat{\mu }_{t_{11},t_{21}} + \hat{\mu }_{t_{21},t_{2j}}\right) \nonumber \\&= \sigma ^2_{t_{11},t_{1i,pooled}} + \sigma ^2_{t_{11},t_{21}} + \sigma ^2_{t_{21},t_{2j,pooled}}. \end{aligned}$$

This is an example of a completely indirect connecting trial. Thus, we have shown that when connecting two arbitrary sub-networks with a single two-arm indirect trial, the variance of any comparative effect size of interest is the sum of the new within-trial variance $\sigma ^2_{t_{11},t_{21}}$ and additional variance parameters that correspond to the individual sub-networks.   

Under the assumption that $\sigma ^2_{t_{11},t_{21}}$ is constant across all possible trials, the best connecting trial will always be direct (i.e., exactly the COI), which is clear from the variance structure presented above. However, in the case of binomial responses, the assumption of constant within-trial variance is not appropriate; instead, we can use information from the existing network to estimate the risk of all of the treatments. This will provide insight on the variance in the new trial. The choice of trial that minimizes a COI is then network dependent, and it may not always be the case that the best connection is direct. Connecting treatments indirectly may result in a lower variance estimate under certain conditions, which we will explore through our real data application.

### Simple example

**Fig. 1:**
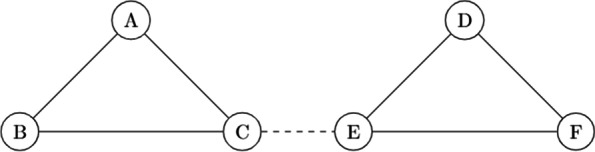
Example network of trials. An example of a disconnected network of trials that has been connected through a new trial. One subnetwork consists of treatments A, B, and C, while the other subnetwork consists of treatments, D,E and F. A trial with treatments C and E connects the two sub-networks, represented by the dashed line

To illustrate a network of trials, we have included an example in Fig. 1 of a network consisting of two disconnected sub-networks, with sub-network one having treatments A, B, and C and sub-network two having treatments D, E, and F. In this case, a new trial has been conducted between treatments C and E to connect sub-networks one and two. If the researchers are originally interested in a comparison between C and E, this is a direct connecting trial, otherwise, it is an indirect connecting trial.

### Proposed procedure

We propose a general selection procedure to find the optimal two-arm trial to connect a disconnected network when the COI is between two disconnected treatments of interest, $c_1$ and $c_2$, from sub-network one and sub-network two, respectively. The steps are as follows:

1. Consider all possible connecting two-arm trials.
2. For each connecting two-arm trial, do the following:
  a Create a new fully connected network consisting of the two previously separated networks and the new trial. Set the new design matrix $\varvec{X}$ such that the overall baseline is the baseline from network one. Append a new row corresponding to the connecting trial.
  b Calculate the within-trial variance $\sigma ^2_{t_1,t_2}$ (based on Eq. 2) as
    $$\begin{aligned} \sigma ^2_{t_1,t_2} = \frac{1}{n_1p_1(1-p_1)}+\frac{1}{n_2p_2(1-p_2)} \end{aligned}$$
    for given treatments $t_1$ and $t_2$. Fix $n_1 = n_2 = n/2$, where *n* is the total sample size in the connecting trial, and set $p_1,p_2$ as the risks of treatments $t_1,t_2$.
  c Use Eq. 11 (or equivalently, Eq. 4) to determine the variance of the COI between treatments $c_1$ and $c_2$ , $\text {Var}(\hat{\mu }_{c_1,c_2})$.
3. Find the optimal connecting two-arm trial that minimizes $\text {Var}(\hat{\mu }_{c_1,c_2})$.

In practice, the risks used in the above selection procedure, $p_1$ and $p_2$, can be estimated from existing data in the following manner:

1. Analyze each network separately using a frequentist based fixed-effects model for NMA.
2. For each sub-network, calculate the risk of all treatments. To do so, obtain estimates of the risks of the baseline treatment, $p_{b}$, from the literature. Then, for any other treatment *t* the risk $p_t$ is calculated as
  12 $$\begin{aligned} p_{t}&= \frac{\frac{p_b}{1-p_b}e^{LOR_{t,b}}}{1+\frac{p_b}{1-p_b}e^{LOR_{t,b}}}\nonumber \\&= \frac{p_b e^{LOR_{t,b}}}{1+p_b e^{LOR_{t,b}}-p_b}, \end{aligned}$$
  where $LOR_{t,b}$ is the estimated log-odds ratio of treatment *t* to baseline *b* from the network meta-analysis conducted in step 1.

## Application and simulation

In this section, we use a real data set to illustrate our procedure for identifying the best connecting two-arm trial based on the methods above. We then conduct a simulation study to confirm our findings and verify the variance formulas we presented. All analysis is performed using R version 4.1.1.

### Real data application procedure

**Fig. 2:**
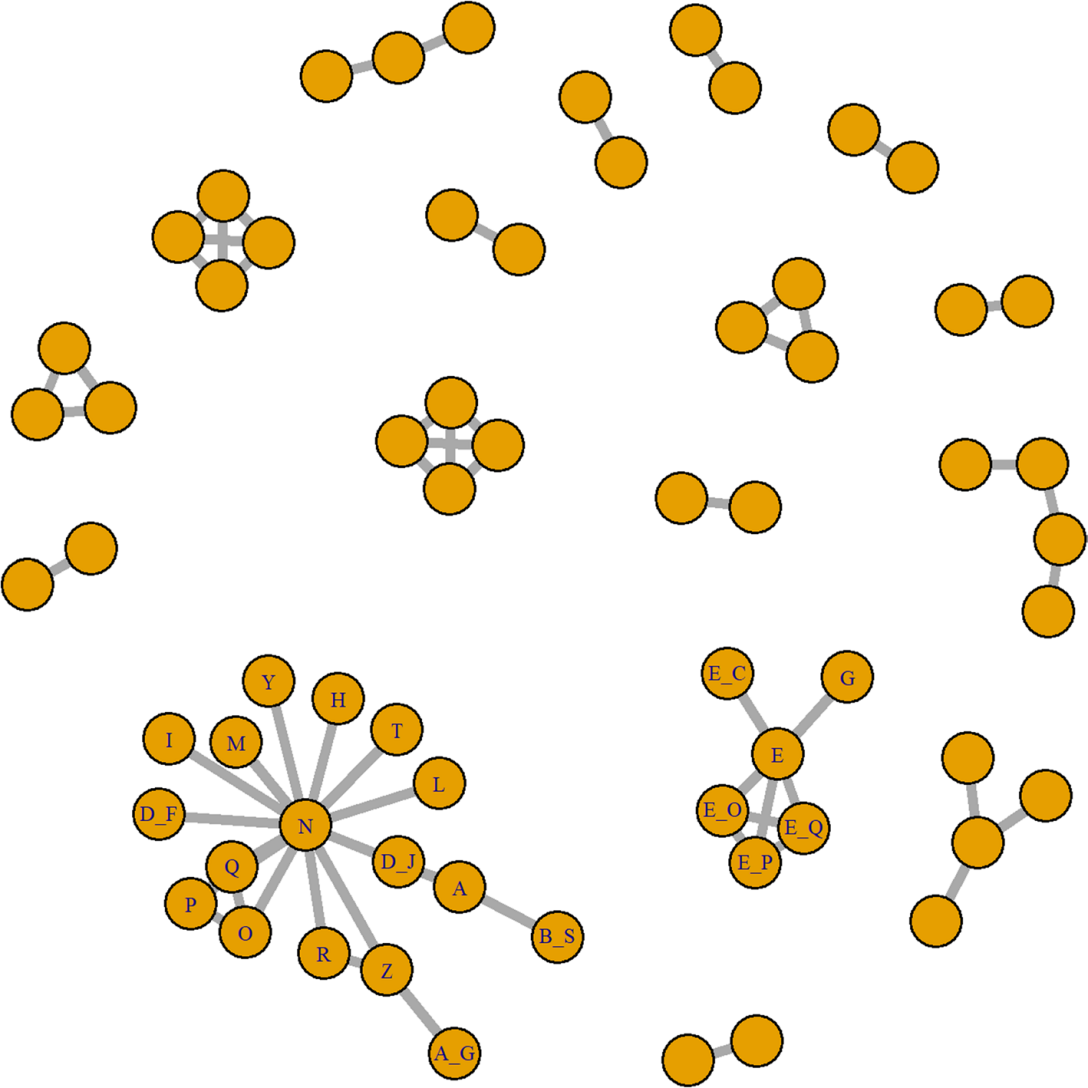
Full network of studies relating to BRD. The entire network of 53 studies, showing the disconnected nature of the studies. Only the treatments that will be used throughout this paper have been labeled

We apply our methods to data from a previously published network meta-analysis on the use of bacterial and viral vaccines for the prevention of bovine respiratory disease (BRD) in beef cattle [15]. A total of 53 studies reported morbidity due to BRD, with the full network shown in Fig. 2. The outcome of interest is an indicator for morbidity and all studies reported log-odds ratios. When conducting a network meta-analysis, the authors focused on the largest sub-network; that is, the authors did not use all of the information related to BRD due to the disconnected nature of the full network. Here, we focus on the two largest sub-networks, which are the two sub-networks with treatments labeled in Fig. 2.

**Fig. 3:**
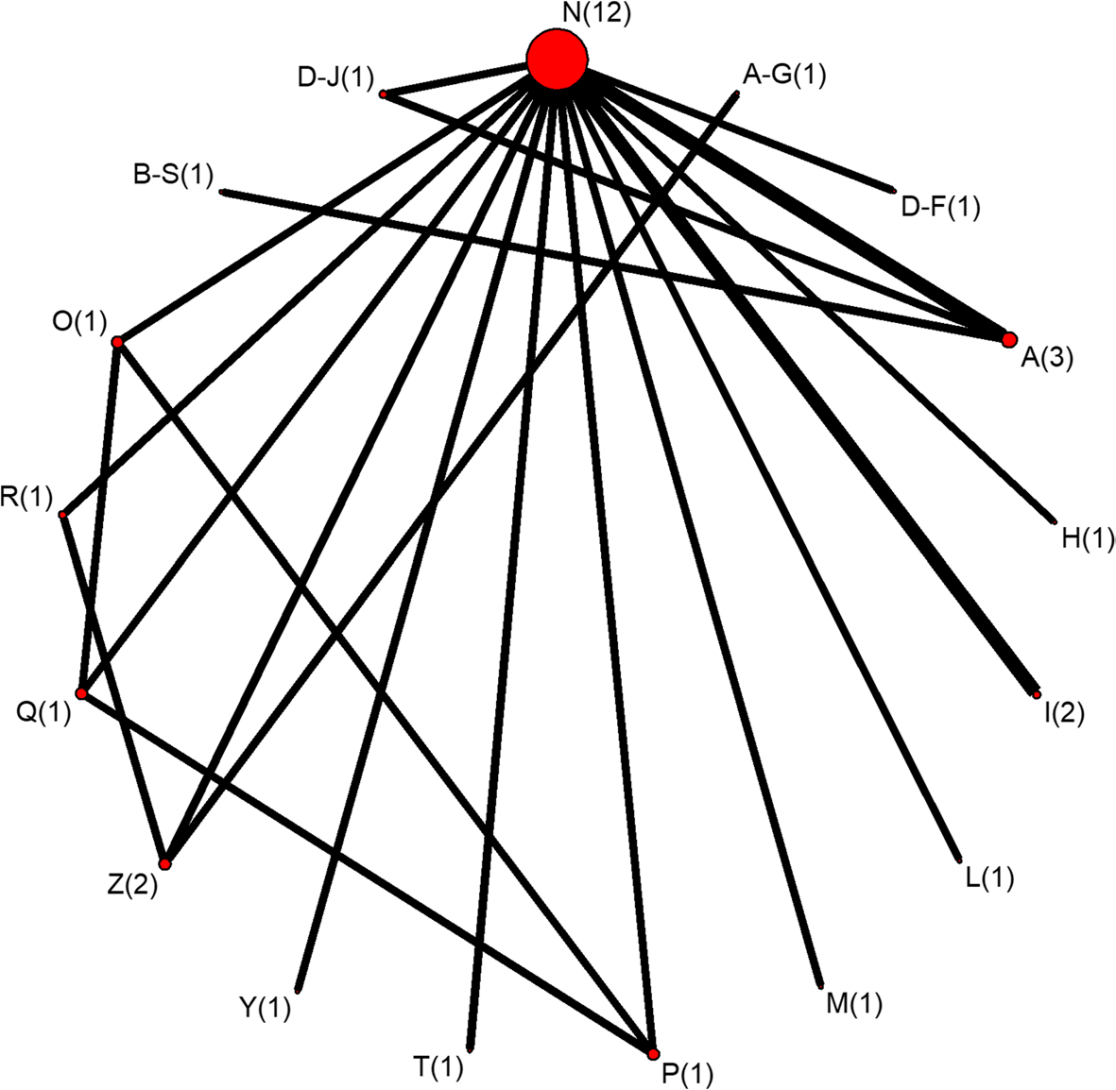
Detailed network plots for sub-network one. The detailed network plot for sub-network one, where the node size corresponds to the total number of studies involving that treatment, and the width of the connecting line corresponds to the number of direct comparisons between treatments

**Fig. 4:**
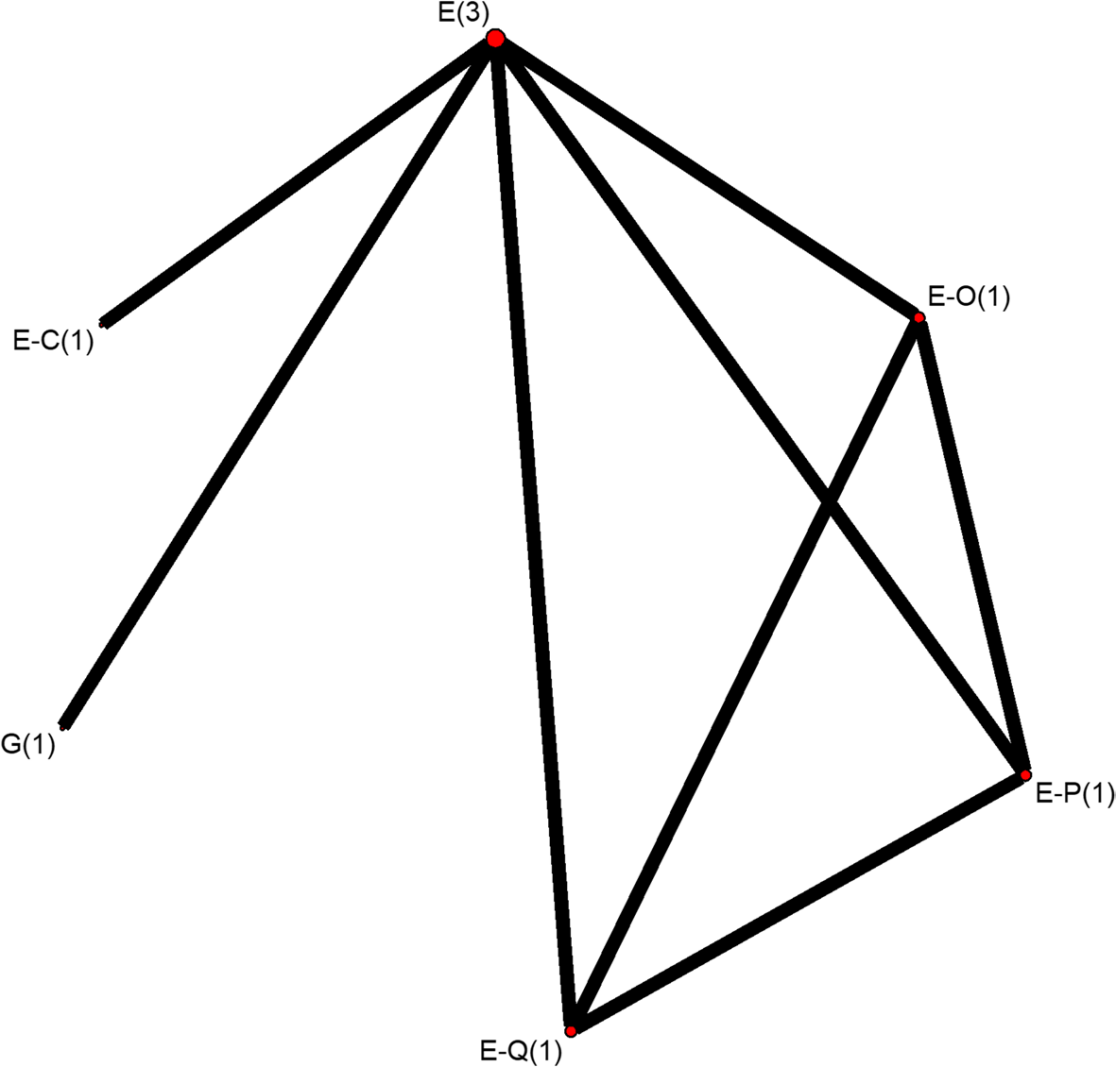
Detailed network plots for sub-network two. The detailed network plot for sub-network two, where the node size corresponds to the total number of studies involving that treatment, and the width of the connecting line corresponds to the number of direct comparisons between treatments

The two sub-networks that are used throughout the remainder of this paper are shown in more detail in Figs. 3 and 4. These two sub-networks will be referred to from this point forward as networks one and two, respectively. Network one includes 17 vaccines from a total of 14 studies. Two of these studies were three-arm trials, one was a four-arm trial, and the remainder were two-arm trials. Network two includes six vaccines from three studies, with one four-arm trial and two two-arm trials.

Suppose that the researchers would like to connect the two largest sub-networks with the goal of estimating a comparison between two vaccines as precisely as possible, for example, the comparison between the two chosen baselines (N and E). Note that these baseline treatments were chosen without loss of generality. Also, we refer to the vaccines as treatments to ensure the language is consistent with the above discussion. Researchers would like to know if it is better to connect the two treatments N and E directly, or if it would be better to leverage information from indirect comparisons and connect the sub-networks elsewhere. To determine the best connecting two-arm trial given a COI, we can apply the proposed selection procedure outlined in the Methods section. In this case, there are a total of 102 (17 x 6) possible two-arm trials.

The results from a network meta-analysis of sub-networks one and two are shown in Tables 1 and 2. We then consider several possibilities for the total sample size of the new connecting trial, *n*, and find the connecting trial such that the variance of $\hat{\mu }_{N,E}$ is minimized (e.g., the COI is between treatments $c_1=N$ and $c_2=E$), as shown in Table 3. This is an example of the COI being the baseline to baseline comparison.

**Table 1:** Estimates of means and standard errors from sub-network one on the log-odds ratio scale, with N as the baseline treatment. Risks are estimated based on Eq. 12

Treatment	$\hat{\mu }_b$	$\sqrt{\text {Var}(\hat{\mu }_b)}$	Estimated Risk
N	-	-	0.3704
A	-0.1710	0.1475	0.3315
A-G	0.1507	0.8317	0.4062
B-S	-0.4294	0.4105	0.2769
D-F	-0.1541	0.3506	0.3353
H	0.0508	0.0754	0.3823
I	-0.4005	0.2946	0.2827
L	-2.7726	1.0992	0.0355
M	-0.4068	0.5774	0.2815
O	0.4055	0.9593	0.4688
P	0.0445	1.0435	0.3801
D-J	0.3900	0.2751	0.4649
Q	0.0445	1.0435	0.3809
R	0.3345	0.7682	0.4511
T	-0.4414	0.3568	0.2745
Y	-0.3589	0.4705	0.2912
Z	0.2692	0.8129	0.4351

**Table 2:** Estimates of means and standard errors from sub-network two on the log-odds ratio scale, with E as the baseline treatment. Risks are estimated based on Eq. 12

Treatment	$\hat{\mu }_b$	$\sqrt{\text {Var}(\hat{\mu }_b)}$	Estimated Risk
E	-	-	0.3831
E-O	-0.2249	0.3220	0.3316
E-P	-0.6267	0.3334	0.2492
E-Q	-0.7243	0.3350	0.2314
G	-0.8122	1.2042	0.2161
E-C	-0.0976	0.0775	0.3604

**Table 3:** The variance in estimation of the comparison of interest, $\text {Var}(\hat{\mu }_{N,E})$, using the best connecting trial when the comparison of interest is N to E compared to the variance using a direct trial of N to E

	$$\text {Var}(\hat{\mu }_{N,E})$$		
*n*	Best Connecting Trial		Direct Trial
6	DJ-E	2.8260	2.8397
8	H-E	2.1221	2.1298
10	H-E	1.6988	1.7038
12	H-E	1.4166	1.4199
14	H-E	1.2151	1.2170
16	H-E	1.0639	1.0649
18	H-E	0.9463	0.9466
20	N-E	0.8519	0.8519
50	N-E	0.3408	0.3408
100	N-E	0.1704	0.1704
200	N-E	0.0852	0.0852
500	N-E	0.0341	0.0341
1000	N-E	0.0170	0.0170

Based on these results, we can see that there are conditions when the best connecting trial is not a direct trial of the two treatments involved in the comparison. Practically, however, there is not a large difference between the variance of the best trial and variance of the direct trial. As the sample size increases, the best connecting trial is the direct trial since as the sample size in the connecting trial increases, so does the precision in the comparative effect size estimate.

Now suppose that researchers are not interested in a baseline-to-baseline comparison of the two sub-networks (this might occur if one of the baselines is no longer a feasible treatment). In the case that the COI is treatments L-G, the best connecting trial is shown in Table 4. In this case, there is a much larger difference between the variance of the best connecting trial and the variance of the direct trial.

**Table 4:** The variance in estimation of the comparison of interest, $\text {Var}(\hat{\mu }_{L,G})$, using the best connecting trial when the comparison of interest is L to G compared to the variance using a direct trial of L to G

	$$\text {Var}(\hat{\mu }_{L,G})$$		
*n*	Best Connecting Trial		Direct Trial
6	DJ-G	4.5914	11.7115
8	H-G	3.7483	8.7837
10	H-G	3.2414	7.0269
12	H-G	2.9035	5.8558
14	H-G	2.6621	5.0192
16	H-G	2.4811	4.3918
18	H-G	2.3403	3.9038
20	N-G	2.2273	3.5135
50	L-G	1.4054	1.4054
100	L-G	0.7027	0.7027
200	L-G	0.3513	0.3513
500	L-G	0.1405	0.1405
1000	L-G	0.0703	0.0703

We further illustrate that it is possible for trials involving completely indirect connections to be better than a direct connection given a COI. Continuing with the COI as L-G, results in Table 5 show that it is possible that a trial involving neither treatment L nor G may be better than a direct trial including both. For a fixed sample size of $n=6$, the best trial that does not include L or G is the trial DJ-E, in which the variance of the COI, $\text {Var}(\hat{\mu }_{L,G}) = 5.4843$. This is smaller than the case when the trial is direct between L-G, in which $\text {Var}(\hat{\mu }_{L,G}) = 11.7115$. Results for additional sample sizes up to $n=20$ are shown in Table 5. These results confirm that it is possible for a trial involving neither of the treatments involved in a COI to be better than a direct connection. The best connecting trial for the the COI L-G is direct with sample sizes $n \ge 50$ and results are the same as those in Table 4.

**Table 5:** The variance in estimation of the comparison of interest, $\text {Var}(\hat{\mu }_{L,G})$ using the best **completely indirect** connecting trial when the comparison of interest is L to G compared to the variance using a direct trial of L to G

	$$\text {Var}(\hat{\mu }_{L,G})$$		
*n*	Best Completely Indirect Connecting Trial		Direct Trial
6	DJ-E	5.4843	11.7115
8	H-E	4.7804	8.7837
10	H-E	4.3571	7.0269
12	H-E	4.0750	5.8558
14	H-E	3.8734	5.0192
16	H-E	3.7222	4.3918
18	H-E	3.6046	3.9038
20	N-E	3.5103	3.5135

As a concluding example, we consider the COI I-EQ. Neither of the treatments involved in this comparison have extreme risks, and this illustrates a case where the difference between the best connecting trial and the direct trial is larger than in the case of N-E. In fact, for $n=6$ this is the COI that results in the largest difference between the best connecting trial and the direct trial (excluding comparisons involving treatment L). Results are shown in Table 6.

**Table 6:** The variance in estimation of the comparison of interest, $\text {Var}(\hat{\mu }_{I,EQ})$, using the best connecting trial when the comparison of interest is I to EQ compared to the variance using a direct trial of I to EQ

	$$\text {Var}(\hat{\mu }_{I,EQ})$$		
*n*	Best Connecting Trial		Direct Trial
6	DJ-E	3.0250	3.5180
8	H-E	2.3211	2.6385
10	H-E	1.8978	2.1108
12	H-E	1.6157	1.7590
14	H-E	1.4141	1.5077
16	I-E	1.2575	1.3193
18	I-E	1.1302	1.1727
20	I-E	1.0284	1.0554
50	I-EQ	0.4222	0.4222
100	I-EQ	0.2111	0.2111
200	I-EQ	0.1055	0.1055
500	I-EQ	0.0422	0.0422
1000	I-EQ	0.0211	0.0211

### Simulation

We extend the ideas presented in the Real data application procedure section to include the simulation of data from new connecting trials to validate our proposed method. We use the following procedure to generate 1000 simulated trials and define the COI to be L-G (that is, $c_1$ = L and $c_2$ = G):

1. Estimate the risks of $p_1$ and $p_2$ as described in the Methods section; that is, estimate the risks of the trial treatments,  t_1 _and t_2_ ,using the existing network data.
2. For each partially indirect connecting two-arm trial that was identified as best (found in Table 4) do the following:
  a Simulate data from the new connecting trial by drawing from both a binomial($n_1$,$p_1$) and binomial($n_2$,$p_2$), where $n_1=n_2=n/2$ and $p_1, p_2$ are the risks of treatments $t_1$,$t_2$ given by Tables 1 and 2. Now we have a simulated number of events for each of the two treatments involved in the new trial, $r_1$ and $r_2$.
  b Estimate $\hat{p}_1 = \frac{r_1}{n_1}$ and $\hat{p}_2 = \frac{r_2}{n_2}$ and use the adjustment to account for proportions of 0,1 in [16].
  c Create a new fully connected network consisting of the two previously separated networks and the new trial. Set the new design matrix $\varvec{X}$ where the overall baseline is treatment N from network one. Append a new row corresponding to the connecting trial.
  d Append a new element to the vector $\varvec{y}$ as $\text {log}(\frac{\hat{p_2}/(1-\hat{p_2})}{\hat{p_1}/(1-\hat{p_1})})$.
  e Calculate the within-trial variance $\sigma ^2_{t_1,t_2}$ as
    $$\begin{aligned} \sigma ^2_{t_1,t_2} = \frac{1}{n_1\hat{p}_1(1-\hat{p_1})}+\frac{1}{n_2\hat{p}_2(1-\hat{p}_2)}, \end{aligned}$$
    for treatments $$t_1$$,$$t_2$$.
  f Use Eq. 4 to calculate the value of $\hat{\mu }_{c_1,c_2}$.
3. Repeat step 2 1000 times and record $\hat{\mu }_{c_1,c_2}$ in each simulated trial.

Results from this simulation are shown in Table 7. Both the bias$(\hat{\mu }_{c_1,c_2})$ and root mean square error, RMSE$(\hat{\mu }_{c_1,c_2})$ are shown. By examining the bias and RMSE in the estimator, we can see that for small sample sizes, a partially indirect trial is preferred to a direct trial. This aligns with the results found in the real data application.

**Table 7:** Simulation of 1000 trials when the COI is L-G

	Best Connecting Trial			Direct Trial	
*n*	Trial	Bias$(\hat{\mu }_{L,G})$	RMSE$(\hat{\mu }_{L,G})$	Bias$(\hat{\mu }_{L,G})$	RMSE$(\hat{\mu }_{L,G})$
6	DJ-G	0.5128	1.3339	-1.5278	2.8869
8	H-G	0.3076	1.1185	-1.4155	2.5219
10	H-G	0.2441	0.9768	-1.3064	2.3086
12	H-G	0.2437	0.9272	-1.2617	2.1308
14	H-G	0.1923	0.8742	-1.1754	1.9721
16	H-G	0.1592	0.8097	-1.1510	1.8554
18	H-G	0.1401	0.8225	-1.0223	1.5928
20	N-G	0.1063	0.7047	-1.0123	1.5995

#### Verification of formulas

We further simulate data to verify the formulas for estimating the variance of a COI presented in the Methods section. As an example, we focus on the case where sub-networks one and two are connected through a two-arm trial involving treatments N and E. Let $\sigma ^2_{t_1,t_2}$ denote the within-study variance of the comparison between treatments $t_1$ and $t_2$, and $\sigma ^2_{t_1,t_2,pooled}$ be the estimate of the variance of the comparative effect size that arises from analysis of the network (i.e., using a fixed effects model for NMA). We verify that Eqs. 4 and 11 produce the same variance estimate. Table 8 shows the breakdown of the variance estimate, which is confirmed using Eq. 4. Table 9 shows a specific example using one data set with a fixed sample size for the connecting trial, $n=1000$. Several comparisons of interest between sub-network one and sub-network two are considered. The results show that when the COI is exactly the connecting trial, the variance of the COI is simply the within-trial variance. Otherwise, the variance is the sum of the new within-trial variance and additional variance parameters that correspond to links in the entire network. These results are consistent with the formulas presented in the Methods section.

**Table 8:** Variance of comparisons of interest across newly connected networks one and two, for all simulated data

COI	Connections (No. of Studies)	Var($\hat{u}_{c_1,c_2}$)
N-E	Direct (1)	$\sigma ^2_{N,E}$
N-EQ	Partially Indirect (1, 1)	$\sigma ^2_{N,E} + \sigma ^2_{E,EQ}$
A-EQ	Completely Indirect (2, 1, 1)	$\sigma ^2_{N,A,pooled} + \sigma ^2_{N,E} + \sigma ^2_{E,EQ}$

**Table 9:** Variance of comparisons of interest across newly connected networks one and two for one simulated data set with $n=1000$

COI	Var($\hat{u}_{c_1,c_2}$)
N-E	0.01716344
N-EQ	0.1293884 = 0.01716344 + 0.112225
A-EQ	0.1511429 = 0.02175445 + 0.01716344 + 0.112225

## Discussion

Evidence from an existing NMA is commonly used to plan a new trial. When a group of trials are connected in a network, several methods have been proposed to identify trial(s) that achieve a desired power or precision for a COI. However, in the case of disconnected networks, there is no literature to guide researchers on how to design a trial to connect any sub-networks. In this paper, we address how to identify connecting trials that minimize the variance of a COI between two disconnected sub-networks. We derive variance formulas which lead to a straightforward computational procedure to identify the best connecting two-arm trial and confirm the results via simulation.

The formulas derived in the Methods section of this paper have several implications. Eq. 11 shows that under a completely indirect connecting trial, the variance of any COI can be written as the sum of three components: a pooled variance from the first sub-network, the within-trial variance from the connecting trial, and a pooled variance from the second sub-network. By writing the variance in this manner, it is clear that the best connecting trial may not always be direct, and is instead network dependent. Further, from Eq. 2, it holds that as $n \xrightarrow {} \infty$, $\sigma ^2_{t_1,t_2} \xrightarrow {} 0$, where $\sigma ^2_{t_1,t_2}$ is the within-trial variance for a trial between treatments $t_1$ and $t_2$. Given a large enough sample size, this implies that the best trial will always be direct. However, the rate of convergence is network dependent, as $\sigma ^2_{t_1,t_2}$ is a function of risks from each sub-network.

Our proposed procedure is a straightforward way for researchers to apply the formulas presented throughout the paper and determine which trial minimizes the variance of a COI. Using a real disconnected network, we have shown that there are several cases when an indirect trial should be preferred to a direct trial. Practically, in the baseline-to-baseline example of N-E, the differences between a direct trial and a partially indirect trial are not very large. In this case, researchers may not be motivated to conduct an indirect trial. However, when the COI is L-G, there is a notable difference between the direct and indirect trial. This difference is evident for both a partially indirect connecting trial and a completely indirect connecting trial. This is due to the extreme risk of treatment L, which makes a direct trial less ideal than an indirect trial in terms of the variance of the COI. Nonetheless, a key takeaway from this paper is exhibited here - in practice, conducting an indirect trial may be preferable to a direct trial when connecting two sub-networks. Further, it may not always be possible to design a direct trial. For example, a feedlot might have an existing contractual obligation to use certain products. Conducting a completely indirect trial would enable the feedlot to obtain an estimate of comparative efficacy of a rival company’s product to make longer term decisions. Similarly, perhaps an older standard of care is the baseline in one sub-network but is expensive to use or has adverse side effects. For example, a long withdrawal for meat consumption will detract from including it as a treatment arm, yet because it is a commonly known treatment stakeholders may still find the comparison to that older standard of care meaningful. Using a partially or completely indirect connecting trial enables such a comparison.

By simulating realizations from both the best (partially indirect) connecting trial and a direct trial of L-G, we have confirmed the conclusions of the real data analysis. Conducting an indirect trial leads to less bias, and a smaller RMSE, than a direct trial, adding more support to our conclusion that the best indirect trial is preferred to a direct trial for certain sample sizes. We note that the small sample size in the connecting trial is contributing to the bias, as discussed in other work [17]. Future simulations may be needed to further validate our proposed procedure.

### Limitations

The methods presented in this paper are limited to use under the assumptions of fixed effects NMA. That is, they can be used under the assumptions of transitivity and consistency. When any of said assumptions are not met, the model needs to be modified and analogous formulas would need to be derived. Further, when designing a trial, researchers may prefer to use a random effects model. The methods presented here do not address that model, but similar results could be derived under a random effect NMA model formulation. In a random effects model, there will be an additional assumption of equal variances. If this assumption is violated then a more general model allowing heterogeneous between-trial variances could be used to derive formulas [18]. The methods here also only consider a single comparison of interest, but in practice researchers may be interested in multiple treatment comparisons. This is a possible extension to our research.

## Conclusion

The goal of this paper is to inform researchers that a direct trial may not always be the best trial to connect sub-networks, and to provide an approach to determine the best trial. In practice, researchers can simplify the procedure by only considering the connecting trials that are of interest to them, rather than all possible connections. With a large enough sample size, the best trial will be the direct trials. However, there are reasons why despite being interested in a particular comparison the feasibility of that comparison may be restricted due to cost, availability and adverse facts, in those situations researchers can use the procedure proposed to find the best connecting trial that does not involve said treatment.

The example used throughout this paper is based on livestock populations, however, the approach proposed is agnostic to this application. The foundation of the method is a valid comparison of NMA networks arising from a systematic review of trials that are reasonably considered to meet the same assumptions for NMA. As such, the approach proposed could be applied to any group trials related to interventions such as biological interventions, pharmaceutical interventions or medical devices. Overall, the purpose of this work is to make it clear that the best connecting trial is network dependent, and this idea is confirmed through both a real data application and simulation.

## Data Availability

We provide the R code and data we used in this paper at https://github.com/lamckeen/Connecting-disconnected-networks.

## Abbreviations

NMA: Network Meta-Analysis
CNMA: Component Network Meta-Analysis
RWE: Real World Evidence
COI: Comparison of Interest
BRD: Bovine Respiratory Disease
